# Exploring the relationship between appendicular skeletal muscle index and urge urinary incontinence risk in adult women: a cross-sectional analysis of the National Health and Nutrition Examination Survey

**DOI:** 10.3389/fnut.2025.1606089

**Published:** 2025-06-18

**Authors:** Jinyu Liu, Kangqiang Weng, Guowei Lin, Huaiding Tang, Jianbing Xie, Lixian Li

**Affiliations:** Department of Urology, The Affiliated Hospital of Putian University, Putian, China

**Keywords:** urge urinary incontinence, urinary incontinence, appendicular skeletal muscle, appendicular skeletal muscle index, sarcopenia, NHANES

## Abstract

**Objective:**

This study aimed to investigate the relationship between the appendicular skeletal muscle index (ASMI) and Urge urinary incontinence (UUI) in a large cohort of adult women.

**Methods:**

This study utilized data from the National Health and Nutrition Examination Survey during the periods of 2001–2006 and 2011–2018. ASMI was identified as the exposure factor. UUI was the outcome variable. We first compared the baseline characteristics of individuals with and without UUI. The effect of ASMI on UUI was assessed using weighted multivariate logistic regression models. Additionally, the relationship between the two was illustrated using restricted cubic splines.

**Results:**

A total of 19,009 women aged 20 and above participated in this study, with 5,960 diagnosed with UUI and 13,049 not affected. The results of the adjusted multivariate logistic regression analysis showed a significant inverse relationship between ASMI and the probability of UUI. Specifically, with every one-unit rise in ASMI, the likelihood of UUI diminished by 69% in the fully adjusted model (OR = 0.31; 95% CI: 0.12–0.82; *p* = 0.02). Furthermore, participants falling within the highest ASMI quartile showed a 28% reduction in UUI risk relative to those in the lowest quartile (OR = 0.72; 95% CI: 0.55–0.94; *p* = 0.02). A restricted cubic spline analysis demonstrated a nonlinear relationship between ASMI and UUI (*p* for nonlinearity = 0.02). Subgroup analyses suggested that various demographic and health factors did not significantly alter this association.

**Conclusion:**

The ASMI was significantly negatively correlated with the risk of UUI, suggesting that an increase in ASMI may have been associated with a lower risk of UUI.

## 1 Introduction

According to the International Continence Society (ICS), urgency urinary incontinence (UUI) was defined as the complaint of involuntary loss of urine associated with urgency (1). UUI was a concerning lower urinary tract condition that affected a diverse range of individuals across all age groups, although it was more prevalent among the elderly (2). Notably, UUI occurred more frequently in women than in men. Previous studies reported that the prevalence of UUI among females in the United States ranged from 9.3 to 30.8% (3). This condition had significant negative impacts on quality of life, leading to psychological distress, limitations on social activities, decreased efficiency in daily living and work, and increased financial burdens, including medical expenses and costs associated with incontinence products (3–7). Treatment options for UUI included behavioral therapy, medications, physical therapy, neuromodulation therapy, and surgical interventions (8, 9). However, the effectiveness of different treatment approaches remained controversial, which may be related to the multifactorial nature of UUI. Preliminary research suggested that UUI was associated not only with age, body mass index (BMI), and socioeconomic status but also with depression and various chronic diseases (3, 10–12). Given the complexity of the underlying mechanisms of UUI, identifying risk factors proved essential for effective management and intervention.

Appendicular skeletal muscle mass (ASM), which referred to the muscle tissue in the upper and lower extremities, was an important indicator for assessing muscle health and served as a foundation for identifying and managing muscle-related issues (13). By monitoring ASM, healthcare professionals could develop effective interventions to improve muscle quality and overall health. However, ASM was closely linked to body size, with greater body weight or height corresponding to higher ASM levels (14). Therefore, in practical applications, the relationship between ASM and height squared or weight squared was more clinically significant, particularly when accounting for individual body size differences (14). The Sarcopenia Project, conducted by the Foundation for the National Institutes of Health (FNIH), utilized extensive and diverse population studies and proposed using ASM/BMI as a novel approach for assessing muscle mass (15), as BMI incorporated both weight and height factors. This index also gained increasing recognition over time. Several studies demonstrated that in older populations, ASM/BMI exhibited significantly stronger correlations with muscle strength, physical performance, and recurrent falls history compared to the conventional ASM/height^2^, which is a traditional and widely-used index for defining low muscle mass (16–18). Cataltepe et al. (19) found that ASM/BMI showed stronger correlations with all physical performance parameters and played a more significant role in improving the diagnosis of sarcopenia. Based on the aforementioned evidence, ASM/BMI emerged as a more robust indicator of functional capacity alterations compared to the conventional sarcopenia index ASM/height^2^. Therefore, in this study, the appendicular skeletal muscle index (ASMI) was formulated as ASM divided by BMI. Nevertheless, the relationship between ASMI and UUI remains unexplored.

It was widely acknowledged that pelvic floor muscles played a significant role in the onset and progression of urinary incontinence (20). While ASMI was derived from ASM, it also served as an indicator of overall muscle health, which included the muscles located in the pelvic area. Furthermore, one study identified a correlation between sarcopenia and overactive bladder (OAB) (21). Based on this research and related theories, it could be proposed that there was a potential link between ASMI and UUI. This study gathered data on adult women from the National Health and Nutrition Examination Survey (NHANES) conducted between 2001 and 2006, as well as from 2011 to 2018, with the aim of assessing the relationship between ASMI and the risk of UUI. The findings intended to provide new insights into the treatment and management of patients experiencing UUI.

## 2 Methods and materials

### 2.1 Study population

**Figure 1:**
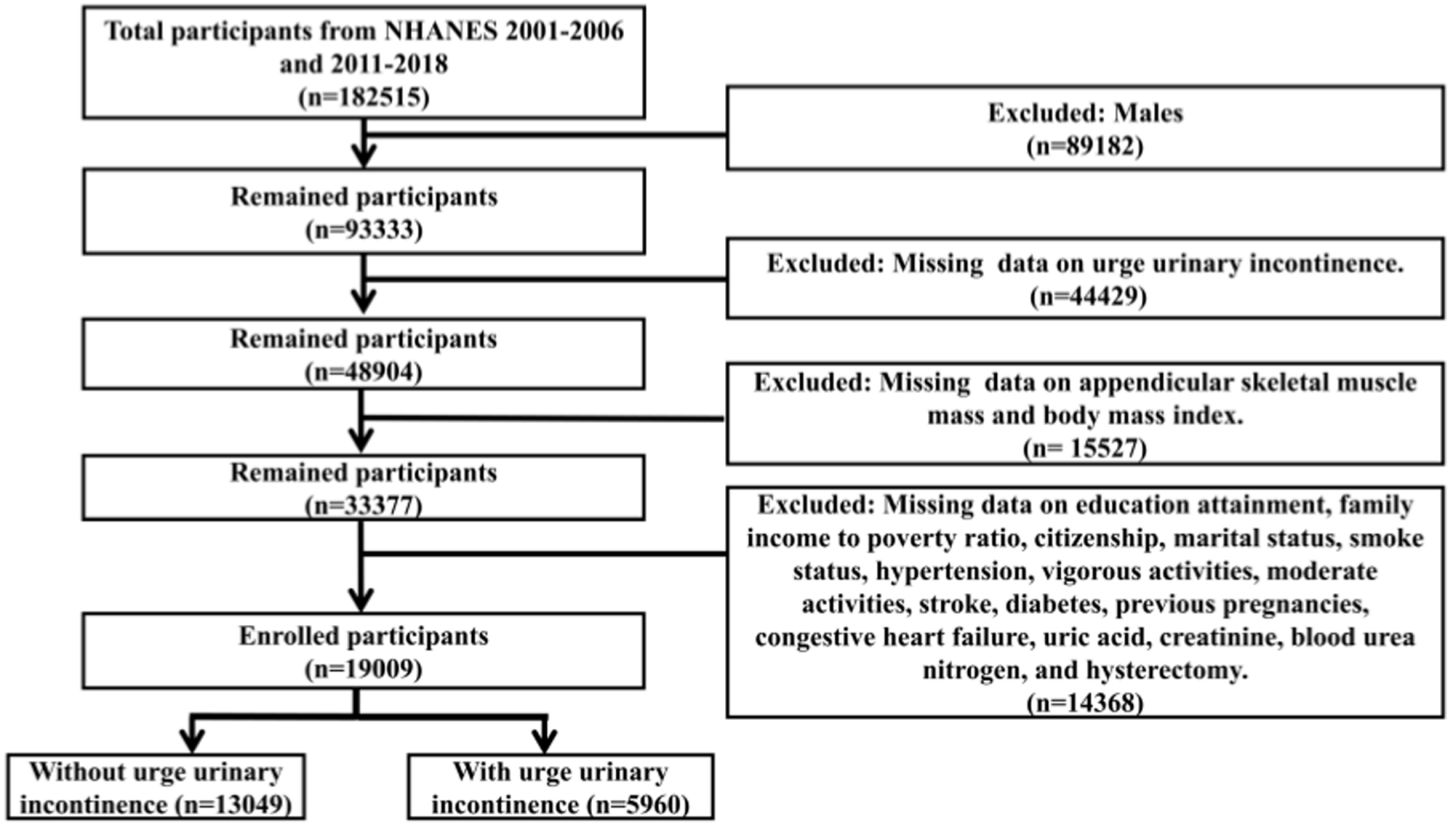
Flowchart for participant selection.

The NHANES was a significant national survey project in the United States, conducted by the National Center for Health Statistics (NCHS) under the Centers for Disease Control and Prevention (CDC). This initiative focused on collecting and analyzing health and nutrition information from individuals across the country, encompassing various health domains, such as chronic diseases, mental health, environmental exposures, and biomarkers. To ensure national representation, NHANES employed a stratified, multistage probability sampling technique. The survey was conducted every 2 years and included participants of varying ages, genders, races, and geographic regions. The sample design accounted for the diversity within the United States, aiming to accurately reflect the health status of different demographic groups. The data collected by NHANES were widely utilized across multiple fields, including the formulation of public health policies, the implementation of health education programs, and scientific research. Researchers and public health officials leveraged this data to identify health issues, assess the effectiveness of health interventions, and develop targeted health policies for specific populations. Furthermore, every participant provided a signed informed consent form, and the study received approval from the National Center for Health Statistics Research Ethics Review Board. This study was an observational study that followed the STROBE (Strengthening the Reporting of Observational Studies in Epidemiology) guidelines to ensure the transparency and consistency of the study design and reporting. This research utilized data obtained from NHANES during the periods of 2001–2006 and 2011–2018. Participants had to be women aged 20 years and older with complete datasets. Additionally, ASM was obtained through dual-energy X-ray absorptiometry (DXA) scans, which have their own exclusion criteria. These criteria include being pregnant, reporting the use of radiographic contrast agents (such as barium) in the past week, and individuals self-reporting a weight greater than 450 pounds or a height over 6 feet 5 inches (limitations of the DXA equipment). Considering these inclusion and exclusion criteria, 19,009 participants were ultimately selected for this study (Figure 1).

### 2.2 Definition of appendicular skeletal muscle index

ASMI was the exposure variable in this study. The calculation formula for ASMI was ASM (kg) divided by BMI (kg/m^2^). The total muscle mass of the arms and legs constitutes ASM. BMI values were directly obtained from the Body Measures section in the Examination Data of NHANES.

### 2.3 Definition of urge urinary incontinence

In this study, UUI served as the primary outcome variable, defined based on the following questionnaire: “Over the last 12 months, have you had any incidents of leaking urine or losing control, feeling a sudden urge to urinate and finding it hard to get to the bathroom quickly?” Individuals who responded “yes” were classified as having UUI, while those who responded “no” were classified as not having UUI.

### 2.4 Assessment of covariates

Drawing from prior research, this study extracted various covariates from the NHANES database. Age was classified into three categories: 20–39 years, 40–59 years, and 60 years and older. Education was segmented into three tiers: individuals with education below high school, those who graduated high school, and those who pursued college or higher education. Race was classified into the following groups: Mexican American, Non-Hispanic Black, Non-Hispanic White, Other Hispanic, and Other Race. The family income to poverty ratio (PIR) was categorized as less than 1.3, between 1.3 and 3.4, and 3.5 or higher. Smoking status was classified into three categories: never smoked (fewer than 100 cigarettes in their lifetime and not currently smoking), former smokers (at least 100 cigarettes in their lifetime but had quit), and current smokers (at least 100 cigarettes in their lifetime and still smoking). Marital status is divided into the following groups: divorced, living with partner, married, never married, separated, and widowed. Diabetes or hypertension was considered present if diagnosed by a healthcare professional or if relevant medications were being used. Vigorous activity was defined as engaging in at least 10 min of intense activity that resulted in significant sweating or a substantial increase in breathing or heart rate. Moderate activity was defined as engaging in at least 10 min of moderate activity that resulted in only slight sweating or a slight to moderate increase in breathing or heart rate. If the participants engaged in vigorous or moderate activity, they answered yes; otherwise, they answered no. The assessment of stroke, congestive heart failure, and hysterectomy, relied on self-report in the Questionnaire, with the question “Has a doctor or other health professional ever informed you of having [respective condition]?” Previous pregnancies were divided into “Yes” and “No” groups. The study accounted for variables including urea nitrogen in the blood, uric acid, and creatinine in serum.

### 2.5 Statistical analysis

In this study, all participants were divided into two groups: those with UUI and those without UUI. Continuous variables were represented by mean ± standard deviation (SD), while categorical variables were expressed as proportions (%) and counts (*n*). Weighted Student’s *t*-tests were used to analyze the differences in continuous variables between the two groups, while the association between categorical variables and the presence of urgency urinary incontinence was assessed using weighted chi-square tests. In this study, we employed two primary effect size indicators, Cohen’s *d* and Cramer’s *V*, to evaluate the relationships between different variables and the magnitude of group differences. Cohen’s *d* was used to measure the standardized effect size of the mean difference between two groups. Based on previous medical literature (22, 23), the effect sizes were categorized as: small (0.2 ≤ *d* < 0.5), medium (0.5 ≤ *d* < 0.8), and large (*d* ≥ 0.8). Cramer’s *V* was employed to assess the strength of association between categorical variables. In accordance with prior medical studies (24, 25), the association strengths were classified as: very strong (>0.25), strong (>0.15), moderate (>0.10), weak (>0.05), and very weak or no association (≤0.05). ASMI was analyzed not only as a continuous variable but also categorized into weighted quartiles. Multiple logistic regression models were used to estimate the relationship between ASMI and UUI, expressed as odds ratios (OR) and corresponding 95% confidence intervals (CI). Model 1 did not include any covariates, Model 2 incorporated covariates including age, race, PIR, marital status, citizenship, and education attainment. Model 3, building on Model 2, added smoking status, hysterectomy, stroke, vigorous activities, diabetes, hypertension, previous pregnancies, urea nitrogen in the blood, moderate activities, congestive heart failure, uric acid, and creatinine in serum. Restricted cubic splines (RCS) were a statistical method used to model nonlinear relationships by flexibly fitting data with different polynomial segments at multiple knots, while maintaining the smoothness and interpretability of the model. This method effectively captured complex nonlinear relationships between variables without easily leading to overfitting. Therefore, in this study, a restricted cubic spline (RCS) curve was employed to illustrate the relationship between ASMI and UUI, based on Model 3, which included all covariates. Subgroup analyses were conducted based on age, educational attainment, PIR, smoking status, vigorous activities, congestive heart failure, and hysterectomy to assess whether these factors influenced the relationship between ASMI and the risk of UUI. All analyses were performed using R software (version 4.2.3). Differences were considered statistically significant when the *p*-value was less than 0.05.

## 3 Results

### 3.1 Demographic characteristics

As shown in the flowchart (Figure 1), a total of 19,009 female adult participants aged 20 and above were enrolled in the final analysis of this study, including 13,049 individuals without UUI and 5,960 individuals with UUI. Table 1 presented the baseline characteristics of the two groups. The study found that, compared to the group without UUI, the group with UUI had a higher proportion of individuals over 60 years old, those who did not engage in vigorous or moderate activities, individuals with a history of stroke, those who had undergone a hysterectomy, as well as those with hypertension and diabetes, citizens, and those with previous pregnancies. In contrast, the proportions of individuals with a college education or higher, those with a PIR of 3.5 or greater, never smokers, and those who were never married were lower. Additionally, the UUI group had higher levels of appendicular skeletal muscle mass, but lower levels of ASMI. Compared to the group without UUI, the group with UUI exhibited higher appendicular skeletal muscle mass but a lower ASMI. The difference in ASMI between the two groups was statistically significant (*p* < 0.05), with a Cohen’s *d* of 0.29.

**Table 1:** Participants’ clinical characteristics obtained from the National Health and Nutrition Examination Survey.

Variables	Without UUI	With UUI	*p*-value	Effect size
	(*n* = 13,049)	(*n* = 5,960)		
Continuous				Cohen’s *d*
Appendicular skeletal muscle (kg)	17.67 ± 0.09	18.10 ± 0.11	<0.001	0.11
BMI (kg/m^2^)	28.32 ± 0.17	30.27 ± 0.20	<0.0001	0.27
Appendicular skeletal muscle index [kg/(kg/m^2^)]	0.63 ± 0.00	0.61 ± 0.00	<0.0001	0.29
Categorical				Cramer’s *V*
Age, *n*(%)			<0.0001	0.20
20–39 years	3,379 (27.95)	631 (12.58)		
40–59 years	5,365 (50.18)	2,419 (48.98)		
≥60 years	4,305 (21.88)	2,910 (38.44)		
Race, *n*(%)			0.09	0.05
Mexican American	2,226 (6.01)	1,049 (5.29)		
Non-Hispanic Black	2,606 (10.33)	1,176 (11.46)		
Non-Hispanic White	6,561 (73.13)	3,266 (75.25)		
Other Hispanic	670 (4.53)	218 (3.87)		
Other Race	986 (6.01)	251 (4.14)		
Educational attainment, *n*(%)			<0.0001	0.08
Below high school level	3,057 (14.30)	1,761 (19.73)		
College level and above	6,941 (61.03)	2,592 (52.64)		
High school level	3,051 (24.67)	1,607 (27.64)		
PIR, *n*(%)			<0.001	0.07
<1.3	3,509 (19.39)	1,943 (24.44)		
1.3–3.4	4,982 (35.59)	2,334 (37.76)		
≥3.5	4,558 (45.03)	1,683 (37.80)		
Smoke status, *n*(%)			0.02	0.04
Former	2,780 (22.20)	1,541 (25.34)		
Never	8,049 (57.59)	3,326 (53.03)		
Current	2,220 (20.21)	1,093 (21.63)		
Vigorous activities, *n*(%)			<0.0001	0.12
No	9,845 (70.84)	5,064 (82.06)		
Yes	3,204 (29.16)	896 (17.94)		
Moderate activities, *n*(%)			<0.0001	0.07
No	6,503 (43.72)	3,482 (51.29)		
Yes	6,546 (56.28)	2,478 (48.71)		
Hysterectomy, *n*(%)			<0.0001	0.14
No	9,172 (71.32)	3,378 (56.88)		
Yes	3,877 (28.68)	2,582 (43.12)		
Stroke, *n*(%)			<0.0001	0.07
No	12,673 (97.36)	5,592 (94.64)		
Yes	376 (2.64)	368 (5.36)		
Diabetes, *n*(%)			<0.0001	0.08
No	11,633 (91.96)	4,919 (87.11)		
Yes	1,416 (8.04)	1,041 (12.89)		
Hypertension, *n*(%)			<0.0001	0.10
No	8,182 (66.92)	3,102 (56.82)		
Yes	4,867 (33.08)	2,858 (43.18)		
Congestive heart failure, *n*(%)			<0.0001	0.11
No	12,773 (98.74)	5,634 (95.04)		
Yes	276 (1.26)	326 (4.96)		
Marital status, *n*(%)			<0.0001	0.13
Divorced	1,569 (12.06)	937 (16.30)		
Living with partner	735 (5.80)	243 (4.13)		
Married	6,835 (58.66)	2,675 (51.65)		
Never married	1,702 (11.96)	567 (9.01)		
Separated	453 (2.55)	228 (2.72)		
Widowed	1,755 (8.98)	1,310 (16.19)		
Citizenship, *n*(%)			<0.001	0.05
No	1,299 (6.01)	403 (3.37)		
Yes	11,750 (93.99)	5,557 (96.63)		
Previous pregnancies, *n*(%)			<0.0001	0.08
No	1,849 (16.09)	547 (9.79)		
Yes	11,200 (83.91)	5,413 (90.21)		
Appendicular skeletal muscle index			<0.0001	0.13
Quantile 1 (0.29, 0.54)	2,909 (17.21)	1,814 (23.35)		
Quantile 2 (0.54, 0.60)	3,130 (23.51)	1,624 (28.03)		
Quantile 3 (0.60, 0.68)	3,328 (27.06)	1,491 (28.92)		
Quantile 4 (0.68, 1.16)	3,682 (32.22)	1,031 (19.71)		

BMI, body mass index; PIR, family income to poverty ratio; UUI, urge urinary incontinence. Data are expressed as means ± SD or number (%). *χ*^2^ test for categorical variables. *t*-test for continuous variables.

### 3.2 Association between ASMI and UUI

**Figure 2:**
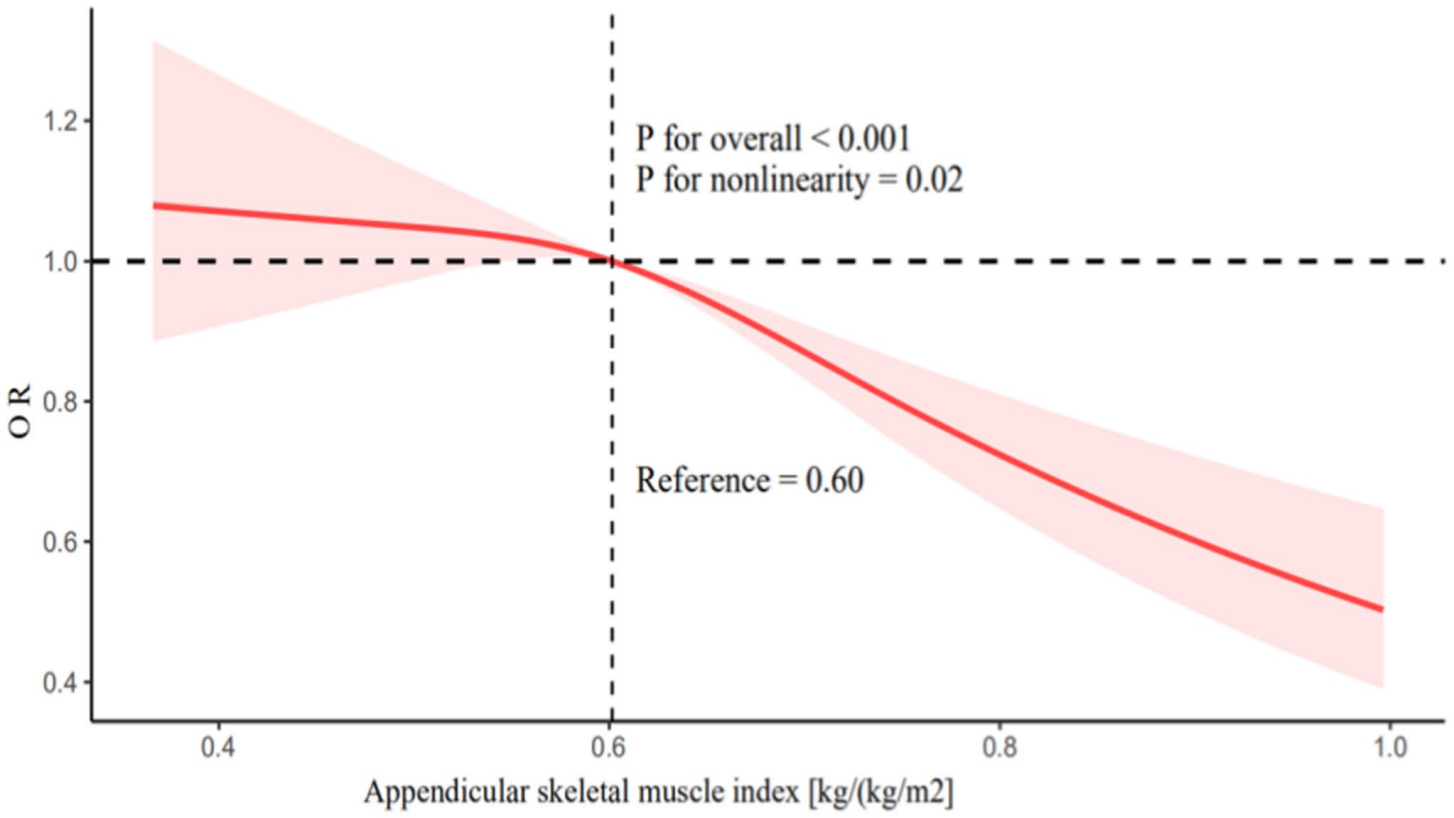
Restricted cubic splines analysis of the association between appendicular skeletal muscle index and urge urinary incontinence. Adjusted for age, race, family income to poverty ratio, marital status, citizenship, education attainment, smoking status, hysterectomy, stroke, vigorous activities, moderate activities, congestive heart failure, diabetes, hypertension, previous pregnancies, blood urea nitrogen, uric acid, and serum creatinine.

To verify the relationship between ASMI and UUI, we conducted logistic regression analysis using three models (Table 2). In Model 1, which did not adjust for any covariates, the risk of UUI decreased by 95% for each unit increase in ASMI (OR = 0.05; 95% CI: 0.02–0.11; *p* < 0.0001). A comparable correlation was noted in Model 2, which accounted for covariates including age, race, PIR, marital status, citizenship, and education attainment. In this model, the risk of UUI decreased by 81% for each unit increase in ASMI (OR = 0.19; 95% CI: 0.07–0.50; *p* = 0.001). Furthermore, Model 3, which includes all covariates, still showed a significant association between ASMI and the risk of UUI. As per the findings of Model 3, an increase of one unit in ASMI was linked to a 69% reduction in the likelihood of UUI (OR = 0.31; 95% CI: 0.12–0.82; *p* = 0.02). Relative to the first quartile of ASMI, being in the fourth quartile reduced the risk of UUI by 28% (OR = 0.72; 95% CI: 0.55–0.94; *p* = 0.02). RCS analysis revealed a nonlinear relationship between ASMI and the risk of UUI, showing a decreasing trend in UUI risk as ASMI increases (Figure 2; *p* for overall <0.001, *p* for nonlinearity = 0.02).

**Table 2:** Association between appendicular skeletal muscle index and urge urinary incontinence.

Exposure	Model 1		Model 2		Model 3	
	OR, 95% CI	*p*	OR, 95% CI	*p*	OR, 95% CI	*p*
Appendicular skeletal muscle index [kg/(kg/m^2^)]						
Continuous	0.05 (0.02, 0.11)	<0.0001	0.19 (0.07, 0.50)	0.001	0.31 (0.12, 0.82)	0.02
Categorical						
Quantile 1 (0.29, 0.54)	Ref		Ref		Ref	
Quantile 2 (0.54, 0.60)	0.88 (0.72, 1.07)	0.20	0.98 (0.79, 1.21)	0.82	1.02 (0.82, 1.27)	0.88
Quantile 3 (0.60, 0.68)	0.79 (0.62, 1.00)	0.05	0.95 (0.73, 1.25)	0.73	1.00 (0.75, 1.33)	1.00
Quantile 4 (0.68, 1.16)	0.45 (0.36, 0.56)	<0.0001	0.64 (0.49, 0.84)	0.001	0.72 (0.55, 0.94)	0.02
*p* for trend		<0.0001		0.002		0.02

Model 1 was adjusted for none. Model 2 was adjusted for age, race, family income to poverty ratio, marital status, citizenship, and education attainment. Model 3 was adjusted for age, race, family income to poverty ratio, marital status, citizenship, education attainment, smoking status, hysterectomy, stroke, vigorous activities, moderate activities, congestive heart failure, diabetes, hypertension, previous pregnancies, blood urea nitrogen, uric acid, and serum creatinine.

### 3.3 Subgroup analysis

Based on stratified analysis considering age, PIR, marital status, citizenship, education attainment, smoking status, race, hysterectomy, stroke, vigorous activities, congestive heart failure, diabetes, hypertension, and previous pregnancies (Table 3). This indicates that identified baseline traits had no effect on altering this association.

**Table 3:** Subgroup analyses of the association between appendicular skeletal muscle index and urge urinary incontinence.

Subgroups	OR (95% CI)	*p*-value	*p* for interaction
Age			0.27
20–39 years	0.08 (0.01, 0.79)	0.03	
40–59 years	0.25 (0.06, 0.96)	0.04	
≥60 years	1.43 (0.17, 11.77)	0.72	
Educational attainment			0.12
Below high school level	0.04 (0.00, 0.33)	0.004	
College level and above	0.69 (0.19, 2.50)	0.57	
High school level	0.10 (0.01, 0.72)	0.02	
PIR			0.49
<1.3	0.07 (0.01, 0.46)	0.01	
1.3–3.4	0.19 (0.04, 0.95)	0.04	
≥3.5	0.89 (0.19, 4.24)	0.88	
Smoke status			0.15
Former	1.47 (0.22, 9.71)	0.69	
Never	0.37 (0.09, 1.52)	0.16	
Current	0.03 (0.00, 0.54)	0.02	
Vigorous activities			0.51
No	0.28 (0.09, 0.89)	0.03	
Yes	0.53 (0.07, 3.79)	0.52	
Hysterectomy			0.87
No	0.33 (0.10, 1.05)	0.06	
Yes	0.29 (0.04, 2.28)	0.23	
Diabetes			0.15
No	0.23 (0.08, 0.66)	0.01	
Yes	2.99 (0.17, 52.26)	0.45	
Hypertension			0.28
No	0.23 (0.08, 0.69)	0.01	
Yes	0.61 (0.10, 3.73)	0.59	
Marital status			0.89
Widowed	0.95 (0.08, 10.99)	0.97	
Married	0.31 (0.07, 1.33)	0.11	
Divorced	0.20 (0.01, 2.96)	0.24	
Never married	0.66 (0.05, 8.15)	0.74	
Separated	0.02 (0.00, 0.99)	0.05	
Living with partner	0.12 (0.00, 5.11)	0.26	
Race			0.86
Non-Hispanic White	0.41 (0.11, 1.46)	0.16	
Mexican American	0.11 (0.01, 0.88)	0.04	
Non-Hispanic Black	0.18 (0.03, 1.04)	0.05	
Other Race	0.99 (0.01, 152.27)	1.00	
Other Hispanic	0.00 (0.00, 0.21)	0.01	
Previous pregnancies			0.83
No	0.31 (0.03, 3.34)	0.33	
Yes	0.29 (0.10, 0.82)	0.02	
Stroke			0.3
No	0.30 (0.11, 0.80)	0.02	
Yes	0.39 (0.00, 81.54)	0.71	
Congestive heart failure			0.66
No	0.30 (0.11, 0.82)	0.02	
Yes	0.02 (0.00, 27.58)	0.27	

OR, odd ratio; CI, confidence interval; PIR, family income to poverty ratio. Adjusted for age, race, family income to poverty ratio, marital status, citizenship, education attainment, smoking status, hysterectomy, stroke, vigorous activities, moderate activities, congestive heart failure, diabetes, hypertension, previous pregnancies, blood urea nitrogen, uric acid, and serum creatinine. When a stratified analysis of an independent variable is performed, the independent variable itself is not adjusted.

### 3.4 Prevalence of low ASMI and UUI according to age changes

As shown in Table 4, our study sample demonstrated a trend of decreasing ASMI with increasing age. According to the FNIH criteria for low ASMI (26), the prevalence of low ASMI in women aged 20–29 was 2.94%, while the prevalence of UUI was 13.28%. In contrast, among women aged 70 and older, the prevalence of low ASMI reached 24.80%, and the prevalence of UUI reached 44.70%. Both the prevalence of low ASMI and UUI exhibited an increasing trend with advancing age.

**Table 4:** Age-specific ASMI values (mean ± SD) and the prevalence [*n*(%)] of low ASMI based on the criteria from the FNIH Sarcopenia Project.

Age (years)	*N*	ASMI (m^2^)	Low ASMI [*n*(%)]	Urge urinary incontinence [*n*(%)]
20–29	1,978	0.68 ± 0.00	67 (2.94%)	255 (13.28%)
30–39	2,032	0.67 ± 0.01	127 (4.78%)	376 (17.35%)
40–49	2,936	0.65 ± 0.00	245 (6.49%)	853 (25.49%)
50–59	4,263	0.62 ± 0.00	587 (11.43%)	1,391 (30.78%)
60–69	4,335	0.59 ± 0.00	916 (14.46%)	1,575 (37.01%)
≥70	1,905	0.57 ± 0.00	965 (24.80%)	1,510 (44.70%)

## 4 Discussion

This study aimed to investigate the relationship between the appendicular skeletal muscle index (ASMI) and urge urinary incontinence (UUI) in a large cohort of 19,009 adult women (aged ≥20 years) using NHANES data on ASM, BMI, and UUI from 2001–2006 and 2011–2018. We found that in Models 1, 2, and 3, there was a negative correlation between ASMI and the likelihood of UUI. RCS analysis indicated a nonlinear association of ASMI with UUI risk, demonstrating a decreasing trend in UUI risk as ASMI rises. Based on stratified analysis considering age, PIR, marital status, citizenship, education attainment, smoking status, race, hysterectomy, stroke, vigorous activities, congestive heart failure, diabetes, hypertension, and previous pregnancies, the effect of ASMI on the risk of UUI is not influenced by these baseline characteristics.

Since the proposal of ASMI as a potential standard for clinically relevant low lean mass (15), its application has increased significantly (27–30). Previous research has demonstrated a correlation between decreased muscle mass and frailty (26, 31), which closely impacts functional ability and quality of life (31, 32). Urinary incontinence often reflects impaired urinary system function, suggesting a potential link between ASMI and urinary incontinence. Erdogan et al. (33) found a strong association between sarcopenia and urinary incontinence; however, their study did not differentiate between stress urinary incontinence (SUI) and UUI, limiting further exploration of these distinct conditions. Similarly, Song et al. (21) identified a relationship between the sarcopenia index and the likelihood of developing overactive bladder (OAB), yet they did not investigate the specific connection between the sarcopenia index and UUI. Given these gaps in the literature, our study aimed to deepen the understanding of the relationship between ASMI and urinary incontinence, particularly focusing on UUI. By addressing these aspects, we contributed valuable insights to the field.

UUI was a common urinary disorder that significantly impacted quality of life and involved complex underlying mechanisms. This condition was associated with disruptions in the nervous system, urothelium, and smooth muscle function (34). Its characteristics included increased spontaneous myogenic activity, fusiform tonic contractions, and characteristic changes in the ultrastructure of smooth muscle (34). Additionally, factors such as abdominal muscles, pelvic floor muscles, and related structures played a crucial role in the development of UUI (33, 35). Research showed that elderly patients with urinary incontinence not only exhibited decreased pelvic muscle mass but also reduced overall muscle mass (36). ASM as a commonly used indicator for assessing overall muscle mass (13), while ASMI served as an important measure for evaluating sarcopenia (16, 29, 30). The association found in this study between ASMI and the risk of UUI may be primarily related to the decrease in pelvic floor muscle quality. Furthermore, it was important to recognize that ASMI reflected the balance between ASM and BMI, with BMI serving as a primary indicator of obesity (37). Individuals with obesity often experienced bladder dysfunction due to the secretion of pro-inflammatory factors (38), which ultimately contributed to urinary incontinence (39). A higher muscle-to-fat ratio was identified as a protective factor against urinary incontinence in women (37). Consequently, ASMI partially reflected the proportions of muscle and fat, thereby influencing the incidence of UUI.

The main strengths of this study are as follows. First, this is the first study to utilize the ASMI to predict the risk of UUI, which has significant clinical implications. Second, the large sample size enhances the representativeness of the findings. However, this study also has several limitations. First, the cross-sectional design of the data prevents us from establishing causal relationships. Additionally, the covariates included in the analysis may not have been exhaustive, potentially affecting the robustness of the results. Third, some of the data on study variables were self-reported, which may introduce recall bias. Overall, even with these limitations, this investigation sheds light on the association between ASMI and UUI, highlighting the importance of future research to delve deeper into the underlying mechanisms in this field.

## 5 Conclusion

This investigation established a significant inverse relationship between the ASMI and the incidence of UUI among adult females. Specifically, higher ASMI values were associated with a marked reduction in UUI risk, even when controlling for various covariates. These findings suggested that maintaining or improving ASMI might help mitigate the risk of UUI in this population. Moreover, the relationship was consistent across different demographic factors, underscoring the robustness of these results. For practical implications, our study highlighted the potential benefits of interventions aimed at enhancing ASMI in reducing UUI risk among women. Future research should adopt a longitudinal design to establish a causal relationship between ASMI and UUI, employing objective measurement techniques to minimize recall bias. Additionally, further studies should explore the underlying mechanisms that may explain the association between ASMI and UUI.

## Data Availability

The datasets presented in this study can be found in online repositories. The names of the repository/repositories and accession number(s) can be found at: liujinyu027@163.com.
